# Development and validation of the Health Education Adherence Scale for Stroke Patients: a cross-sectional study

**DOI:** 10.1186/s12883-022-02597-2

**Published:** 2022-02-28

**Authors:** Weiwei Ding, Junya Chen, Jing Liu, Beibei Lin, Shihen Li, Fengzhen Li, Junyi Guo, Yun Li, Jufang Li

**Affiliations:** 1 School of Nursing, Wenzhou Medical University, Wenzhou, Zhejiang China; 2 The Second Affiliated Hospital of Wenzhou Medical University, Wenzhou, Zhejiang China; 3 The First Affiliated Hospital of Wenzhou Medical University, Wenzhou, Zhejiang China

**Keywords:** Stroke, Health education adherence, Item reduction process, Item analysis, Exploratory factor analysis, Confirmatory factor analysis, Factor structure, Reliability, Validity, Nursing

## Abstract

**Background:**

Due to the lack of health education adherence assessment tools for stroke patients, the assessment of health education adherence in this population is insufficient, which hinders the prevention and rehabilitation of stroke. This study aims to develop and validate a Health Education Adherence Scale for Stroke Patients (HEAS-SP).

**Methods:**

A cross-sectional design with a purposive sampling method was used for this study. Six hundred and fifty-four eligible participants completed the demographic questionnaire and the HEAS-SP. The data collection lasted for 7 months, from March 1stto September 30th in 2019. Item analysis and exploratory and confirmatory factor analysis were employed to develop and validate the HEAS-SP.

**Results:**

The item analysis, exploratory and confirmatory factor analysis resulted in a 20-item HEAS-SP with 4 domains: medication adherence, diet adherence, rehabilitation exercise adherence, and healthy lifestyle adherence. The four-domain model demonstrated acceptable model fit indexes and the 20-item HEAS-SP demonstrated acceptable reliability and validity.

**Conclusion:**

The 20-item HEAS-SP was shown to have acceptable reliability and validity for assessing health education adherence with respect to diet, medication, rehabilitation exercise and healthy lifestyle in stroke patients, making it a potential basis for developing targeted interventions for stroke patients.

## Background

Stroke is an acute cerebrovascular disease that is characterized by impaired nerve function, limb dysfunction and stroke-related neuropsychiatric sequelae, such as depression, anxiety or apathy [1]. Stroke affects 15 million people worldwide each year, of which 5 million die and another 5 million are permanently disabled. The “four high” characteristics of stroke—high morbidity, high mortality, high disability and high recurrence—make stroke the second leading cause of death in the world and the leading cause of death in China [2]. Since nearly half of stroke-related deaths are caused by the poor management of modifiable risk factors [3], there is now a general consensus that managing stroke risk factors can significantly improve disease outcomes [4]. Health education regarding medication, diet, rehabilitation exercise and healthy lifestyle is an important method for risk factor management [4], which can improve the recovery of limb function, reduce disability, alleviate depression and improve long-term quality of life in stroke patients [5, 6]. However, even in developed countries, approximately 50% of patients exhibited poor adherence to health education, and the situation is even worse in developing countries [7]. Poor health education adherence on medication may hinder secondary prevention of stroke and is the main cause of increased recurrence of stroke [8] and good adherence to medication may improve stroke patients’ clinical outcomes [9]; furthermore, health education adherence to healthy diet may help to prevent stroke [10]; in addition, increased adherence to rehabilitation exercise was associated with improved functional recovery after mild to moderate stroke [11] and also helped to promote the management of residual deficits of stroke patients [12]; and health education adherence on healthy lifestyle is essential to improve the primary stroke prevention and functional outcomes of stroke patients [13, 14]. Thus, enhancing the health education adherence on diet, medication, rehabilitation exercise and healthy lifestyle of stroke patients is an important strategy to improve their functional rehabilitation and prognosis. However, the lack of screening tools for evaluating the whole aspects of health education adherence in stroke patients hinders the assessment and subsequent improvement of health education adherence in this population.

Health education adherence refers to the extent to which patients follow clinical and institutional health education measures regarding the diet, medication use and lifestyle changes or the adoption of behaviors to main health [15]. Presently, the health education adherence of stroke patients has not been fully evaluated due to the lack of specific screening tools. Though some aspects of stroke patients’ health education adherence were evaluated by certain screening tools, there may be some shortcomings. For example, stroke patients’ health education adherence on medication is often assessed by the Morisky Medication Adherence Scale (MMAS-8) [16–18]. However, it is not specifically designed for stroke patients, which may result in lower specificity and sensitivity when used in stroke population. Specifically, items regarding the specific medication, such as the ASA (hypertensive drug—lipid-lowering drug—anticoagulant drug), which were frequently taken by stroke patients is lacking; also, items regarding blood pressure which is an important indicator when taking stroke related medication is also lacking. The lacking of the above components may incur inaccuracy assessment of medication adherence in stroke patients. Furthermore, stroke patients’ health education adherence on rehabilitation exercise has been assessed by the Questionnaire of Exercise Adherence (EAQ) [19]. However, the EAQ is for community stroke patients only and may not be suitable for all stroke patients. In our opinion, there are differences in adherence to exercise between inpatients and community patients: inpatient complete exercise under the supervision of medical staff while community patients are unsupervised, so community patients’ adherence to exercise is relatively poor than inpatients. In addition, the assessment of health education adherence with respect to diet and healthy lifestyle remains unaddressed for stroke patients in the literature. Therefore, it is necessary to develop a specific health education adherence scale for stroke patients that include the four aspects of health education for patients with chronic diseases, namely, diet, medication, rehabilitation exercise and healthy lifestyle [20]. Our research team has developed a Health Education Adherence Scale for Stroke Patients (HEAS-SP) and initially validated it by a two round Delphi expert panel including 21 experts (18 are clinical nursing staff, 1 is stroke health manager and 2 were university nursing teachers), and it demonstrated good content validity in the Delphi expert panel [21]. However, it is still necessary to further validate and explore the construct validity and reliability of the HEAS-SP through rigorous statistical methods.

## Methods

### Aim design and settings

This study sought to further develop, tool reduction and validate the HEAS-SP using item analysis and exploratory and confirmatory factor analysis, which aimed to provide a useful tool for health education professionals to support the formulation of targeted health education intervention strategies. A cross-sectional design with a purposive sampling method was employed to collect data from one general hospital located in Southeast China.

### Participants

A cross-sectional study with purposive sampling was used to collect data from eligible participants who were admitted to the neurology wards at one comprehensive hospital in Southeast China. The data collection hospital has a total of 3380 beds and has more than 200 beds in 5 neurology wards. Patients aged 18 years or older who were diagnosed with stroke, were ill for more than one week and consented to participate were eligible for the study. Patients with aphasia or attention disorders, patients whose cognition was severely impaired according to the Mini-Mental State Examination score (MMSE score ˂10), patients with mental disorders, and patients who were seriously ill and needed critical care were excluded (patients who were transferred from the critical care to general ward can be considered for inclusion). We determined the sample size based on the number of items on the HEAS-SP and the use of factor analysis in this study. As proposed by Rouquette and Falissard [22], the number of expected participants should be 5 to 10 times the number of items on the questionnaire when performing an exploratory factor analysis (EFA). The HEAS-SP had 34 items; thus, an EFA sample size of 170 to 340 was required. Assuming that 20% of the questionnaires would be invalid, 213–425 participants would be needed. Furthermore, the sample size for the confirmatory factor analysis (CFA) should be 5 to 10 times the freely estimated parameters in the confirmatory model [22]. The number of freely estimated parameters in the confirmatory model could not yet be determined, so we aimed for a CFA sample size that was greater than that for the EFA. Ultimately, we collected data from 654 participants who were eligible for data analysis. Of the 654 participants who completed the survey, 60 were selected to complete the survey again two weeks later to examine the test–retest reliability of the HEAS-SP.

### Procedure

Potential participants were selected by research team members, who reviewed their medical records to determine whether they met the inclusion criteria or not. Eligible participants were invited to take a survey that lasted for approximately 30 min on the hospital ward. The questionnaires were handed out to the stroke patients in face-to-face interviews. Then, the participants completed the questionnaires by themselves. However, if the participants had difficulty completing the questionnaires due to a low educational level, the researchers would read the questionnaire items to the participants and record the responses. The questionnaires were taken back and checked immediately after completion. If there were missing answers, the respondents were asked to supplement immediately to ensure no missing answers in the questionnaire. All the study researchers were trained to ensure consistency and accuracy. Specifically, they were acquainted with the study purpose and the questionnaire instructions, which ensured that the study researchers used the same guidance for all participants. Over a period of 7 months, from March 1st to September 30th in 2019, we invited 656 eligible participants, and 654 of them completed the questionnaires. The other two participants withdrew from the study for personal reasons.

### Instruments

A demographic questionnaire and the HEAS-SP were used to collect data. The demographic questionnaire was developed by the researchers based on the literature review. It included the participants’ age, sex, marital status, monthly household income, working status, place of residence, main caregivers, educational level, and medical payment method.

The 34-item HEAS-SP was developed by our research team using a Delphi expert panel [21]. The initial items of HEAS-SP were developed by a literature review, a semi-structured interview with the medical staff and the stroke patients. After a two-round Delphi, the items of the HEAS-SP were decreased from 39 to 34. The 34-itemHEAS-SP was designed to assess the health education adherence of stroke patients. The self-rated scale assesses the four dimensions of stroke patients’ health education adherence: diet (8 items), medication (8 items), rehabilitation exercise (7 items) and healthy lifestyle (11 items). Responses to the items range from 1 to 5 and correspond to “no adherence”, “little adherence”, “occasional adherence”, “frequent adherence”, and “full adherence”. The total score of the scale is calculated from the sum of all the items, and the domain scores are the sums of the items in each dimension. Higher domain and total score of the HEAS-SP indicate higher adherence of stroke patients in total and each domain. In the previous Delphi study [21] the expert panel scoring demonstrated good content validity, as evidenced by the satisfactory item-level content validity index (I-CVI) of 0.67–1.00 and the average scale-level content validity index (S-CVI/Ave) of 1.00.

### Data analysis

IBM SPSS 24.0 and AMOS 24.0 were used to analyze the data. The specific statistical methods performed in this study were descriptive analyses, item analyses, EFA, CFA and reliability analysis. The statistical significance level was *p* < 0.05.

Descriptive statistics were performed to analyze the participants’ demographic data and HEAS-SP domain scores and total scores. Specifically, frequencies and percentages were used to describe the categorical variables, and means and standard deviations were used to describe the continuous variables.

The item analysis included four statistical methods (critical ratio, correlation, Cronbach’s α, and factor analysis) with a series of five indicators: critical ratio, item-total correlation, corrected item-total correlation, Cronbach’s α when an item was deleted, and factor loadings. The criteria for retaining items were as follows: critical ratio ≥ 3.0, item-total correlations and corrected item-total correlations** ≥ **0.40, a decrease in the Cronbach’s α value when an item was deleted, and factor loadings ≥ 0.45 [23]. Items were considered for deletion if they failed to meet three or more item analysis indicators.

The data from the 654 participants were randomly divided into two datasets using the SPSS select cases option. The participants of the two datasets were 323 and 331 respectively, and there were no significant difference between the two datasets regarding the demographic characteristics of the participants. The dataset with 323 participants was subjected to EFA to explore the factor structure of the remaining items. The extraction method was principal component analysis (PCA), and the rotation method was varimax rotation. Varimax rotation is suggested when items are supposed to be independent. Bartlett’s test of sphericity and the Kaiser–Meyer–Olkin (KMO) index were used to confirm the suitability of the sample for EFA, and the KMO greater than 0.60 indicated its suitability for factor analysis [23]. Items with significant factor loadings that were equal to or greater than 0.45 were considered for retention; otherwise, they were deleted [24].Factors with eigenvalues˃1 constituted the scale’s factor structure, and factor numbers were further confirmed with a scree plot [23, 25]. Each selected individual factor should explain at least 5% of the total variance in health education adherence, and all the selected factors should together explain at least 60% of the variance [23].

Then a CFA was run with the data from the group of 331 participants to further establish the factor structure of the remaining items [26]. Three types of model fit indexes were employed to determine the model fit: absolute indexes, relative indexes and parsimony indexes. Absolute indexes employed were the root mean square residual(SRMR), root-mean-square error of approximation (RMSEA), goodness of fit index (GFI) and adjusted GFI (AGFI); relative indexes employed were the normed fit index (NFI), incremental fit index (IFI), Tucker-Lewis index (TLI), and comparative fit index (CFI); parsimony indexes employed were the parsimony goodness-of-fit index (PGFI), parsimony adjusted NFI (PNFI), parsimony comparative fit index (PCFI) and chi-square value/degrees of freedom (CMIN/DF). A good model fit was evidenced by the SRMR value < 0.05; RMSEA value < 0.08; GFI and AGFI values > 0.80 [27]; NFI, IFI, TLI and CFI values ≥ 0.90; PGFI, PNFI and PCFI values > 0.50; and CMIN/DF value between 2.0 and 5.0 [28].

The Cronbach’s α, corrected item-total correlation and item-subscale correlation values were used to test the internal reliability of the final version of the HEAS-SP. The Cronbach’s α value ≥ 0.70, the corrected item-total correlation value ≥ 0.40, and the item-subscale correlation value ≥ 0.40 indicate good internal reliability [28]. Pearson correlation coefficient was used to evaluate the test–retest reliability of the final version HEAS-SP, and the coefficient ≥ 0.70 indicated acceptable test–retest reliability [29].

The composite reliability (CR) and the average variance extracted (AVE) were used to evaluate the convergent validity of HEAS-SP; CR ≥ 0.70 and AVE ≥ 0.50 indicate acceptable convergent validity [30]. The discriminant validity of HEAS-SP was assessed by the comparison between square root of AVE and correlations between domains; the square root of AVE of each domain > the correlation coefficient of that domain and other domains is evidence of acceptable discriminant validity [31].

## Results

### Participant demographics

Six hundred and fifty five participants completed the questionnaires and all of their data were subjected for data analysis. The participants’ mean age was 65.5 years, with a standard deviation of 10.3. Most of the participants were male (62.4%), were married (83.8%), had a high monthly household income (64.4%), and were primarily cared for by family members (76.1%). Almost half of the participants (44.0%) were still working. More than half of the participants lived in rural China (54.4%) and had received more than 6 years of education (56.1%). Most of the patients had medical insurance (96.8%, Table 1).

**Table 1:** Demographics of the participants (*N* = 654)

Variable	Mean	SD	N	%
Age	65.5	10.3		
Male			408	62.4
Female			246	37.6
Married			548	83.8
Participants with high monthly household income (more than 5000RMB equals 706 US dollars)			421	64.4
Employed			288	44.0
Resident in rural China			356	54.4
Cared for by family members			498	76.1
Educated more than 6 years			367	56.1
Have medical insurance			633	96.8

*RMB* Chinese currency

Item analysis of the 34-item HEAS-SP.

All items were qualified for inclusion based on the critical ratio, and Cronbach’s α if item deleted. The item analysis indicators that did not meet the criteria are highlighted in bold (Table 2). Specifically, items A1 and A2 were not qualified on the item-total correlation, corrected item-total correlation and factor loading values; and items D2 and D3 were not qualified on the corrected item-total correlation and factor loading values. Finally, items A1 and A2, which were not qualified on three indicators, were removed; items D2 and D3 were kept to the next step for only not qualified on two indicators. The HEAS-SP items were reduced to 32 in this step.

**Table 2:** Item analysis results for all items (*n* = 654)

Domains/Items	Indicator 1: CR ≥ 3.0	Indicator 2: Item-total correlation ≥ 0.40	Cronbach’s α		Indicator 5: Item factor loadings on their own domians ≥ 0.45	Number of unqualified indicators	Item selection
			**Indicator 3: Corrected item-total correlation ≥ 0.40**	**Indicator 4: Cronbach’s α if item deleted ≤ 0.925**			
**Diet Adherence**							
** A1**. I can adhere to the nurses’ health education to control the type of eating according to my swallowing condition (e.g., paste diet, liquid diet, semi-liquid diet)	8.993	**0.330**	**0.286**	0.925	**0.306**	3	Removed
** A2.** I can adhere to the nurses’ health education to carry out simple swallowing training before eating (stretching the head and neck forward and turning the head to the healthy side)	10.251	**0.380**	**0.333**	0.924	**0.354**	3	Removed
** A3.** I can adhere to the nurses’ health education to pay attention to my eating method	13.052	0.501	0.466	0.923	0.503	0	Selected
** A4.** I can adhere to the nurses’ health education to control my daily salt intake within 6 g (size of a beer cap)	13.465	0.528	0.494	0.923	0.540	0	Selected
** A5.** I can adhere to the nurses’ health education to eat a low-oil diet (no fried foods; the daily oil consumption is 30 g: the amount of oil held by a porcelain spoon)	13.672	0.530	0.497	0.923	0.547	0	Selected
** A6.** I can adhere to the nurses’ health education to eat low-cholesterol foods (excluding animal internal organs, fried foods)	14.304	0.537	0.503	0.922	0.553	0	Selected
** A7.** I can adhere to the nurses’ health education to actively learn the dietary content (such as low-salt, low-fat diet content)	13.343	0.526	0.489	0.923	0.530	0	Selected
** A8.** I can adhere to the nurses’ health education to control the amount of food I eat	13.421	0.501	0.456	0.923	0.497	0	Selected
**Medication Adherence**							
** B1.** I can adhere to the nurses’ health education to take my medicine regularly as prescribed	12.334	0.538	0.500	0.922	0.541	0	Selected
** B2.** I can adhere to the nurses’ health education to take the appropriate dose	12.553	0.547	0.510	0.922	0.552	0	Selected
** B3.** I can adhere to the nurses’ health education to take the medicine on time	13.729	0.577	0.541	0.922	0.581	0	Selected
** B4.** I can adhere to the nurses’ health education to not change the medicine-taking method	13.374	0.564	0.527	0.922	0.567	0	Selected
** B5.** I can adhere to the nurses’ health education to follow the prescription and not change the type of drug	13.205	0.514	0.465	0.923	0.508	0	Selected
** B6.** I can adhere to the nurses’ health education to pay attention to any adverse reactions after taking the medicine	14.484	0.548	0.502	0.922	0.535	0	Selected
** B7.** I can adhere to the nurses’ health education to follow the ASA (hypertensive drug—lipid-lowering drug—anticoagulant drug) drug treatment strategy	16.221	0.563	0.520	0.922	0.558	0	Selected
** B8.** I can adhere to the nurses’ health education to pay attention to my blood pressure when taking medicine	18.661	0.631	0.593	0.921	0.640	0	Selected
**Rehabilitation Exercise Adherence**							
** C1.** I can adhere to the nurses’ health education to exercise on time every day	20.216	0.639	0.605	0.921	0.660	0	Selected
** C2**. I can adhere to the nurses’ health education to do early postural adjustments every day	21.221	0.657	0.624	0.921	0.678	0	Selected
** C3.** I can adhere to the nurses’ health education to stop the rehabilitation exercise when I feel uncomfortable	22.954	0.681	0.649	0.921	0.698	0	Selected
** C4**. I can adhere to the nurses’ health education to not stop the rehabilitation exercise program casually	18.064	0.619	0.582	0.921	0.636	0	Selected
** C5.** I can adhere to the nurses’ health education to practice good limb positioning (passive training 2 times a day, 20 min at a time)	20.627	0.638	0.603	0.921	0.653	0	Selected
** C6.** I can adhere to the nurses’ health education to practice simple vocal exercises according to the clinical education guidance	20.738	0.627	0.590	0.921	0.632	0	Selected
** C7.** I can adhere to the nurses’ health education to practice orientation training (regarding place, time)	16.394	0.556	0.511	0.922	0.5553	0	Selected
**Healthy Lifestyle Adherence**							
** D1.** I can adhere to the nurses’ health education to change my lifestyle after I get sick	12.308	0.488	0.442	0.923	0.479	0	Selected
** D2.** I can adhere to the nurses’ health education to stop bad habits (such as smoking, drinking)	10.240	0.421	**0.374**	0.924	**0.406**	2	Selected
** D3.** I can adhere to the nurses’ health education to do rehabilitation exercises in the community or hospital on time	9.063	0.401	**0.350**	0.924	**0.374**	2	Selected
** D4.** I can adhere to the nurses’ health education to master stroke-related risk factors (such as hypertension, diabetes, hyperlipidemia)	14.448	0.534	0.491	0.923	0.522	0	Selected
** D5.** I can adhere to the nurses’ health education to do physical activity every day for at least 5 days a week, 30–45 min at a time	15.248	0.563	0.523	0.922	0.564	0	Selected
** D6.** I can adhere to the nurses’ health education to adhere to good life habits	12.880	0.506	0.464	0.923	0.498	0	Selected
** D7.** I can adhere to the nurses’ health education to control my emotions	13.863	0.514	0.469	0.923	0.503	0	Selected
** D8.** I can adhere to the nurses’ health education to follow up once every two weeks	13.403	0.498	0.453	0.923	0.489	0	Selected
** D9.** I can adhere to the nurses’ health education to control my blood pressure below 130/80 mmHg (for those with diabetes or hyperlipidemia) or to control my blood pressure at 140/90 mmHg (for those with a history of hypertension)	15.176	0.536	0.494	0.922	0.530	0	Selected
** D10.** I can adhere to the nurses’ health education to regularly monitor my glycated hemoglobin	12.809	0.513	0.469	0.923	0.501	0	Selected
** D11.** I can adhere to the nurses’ health education to control my waist circumference	14.642	0.528	0.487	0.923	0.524	0	Selected

### EFA of the 32-item HEAS-SP

The KMO of 0.903 and the significant Bartlett’s test of sphericity (Chi-square = 6493.206, *p* < 0.001) for the sample of 323 participants confirmed the suitability for factor analysis. An EFA was repeatedly run to obtain the final factor structure of the HEAS-SP. After 4 rounds of EFA, eight items were deleted in sequence: B8, D3, D4, A8, D10, B6, B7, and D5 for various reasons. Thus, the number of HEAS-SP items was decreased to 24. The KMO of 0.901 and the significant Bartlett’s test of sphericity (Chi-square = 5457.152, *p* < 0.001) guaranteed the appropriateness of the final round of EFA. The final round yielded a four-factor structure model with eigenvalues ˃1, and the four factor structure model was further confirmed by the scree plot where there are four dot on the “break point” line. The rotated component matrix revealed that all the items significantly loaded on the selected factors, and the factor loadings of the items were all ≥ 0.40. The four factors of the 24-item HEAS-SP were “diet adherence”, with five items that had a factor loading range of 0.69—0.87; “medication adherence”, with five items that had a factor loading range of 0.67—0.94; “rehabilitation exercise adherence”, with seven items that had a factor loading range of 0.70—0.81; and “healthy lifestyle adherence”, with seven items that had a factor loading range of 0.52—0.66.The variance explained by the four factors was 19.2%, 16.8%, 14.8% and 13.6% for the rehabilitation exercise adherence domain, medication adherence domain, diet adherence domain and healthy lifestyle adherence domain, respectively. The total variance explained by these four factors was 64.3%, suggesting that the retained factors explained enough total variance of health education adherence (Table 3).

**Table 3:** Rotated component matrix of the EFA (*N* = 323)

	**Components**			
	**Factor 1 Rehabilitation exercise adherence**	**Factor 2 Medication adherence**	**Factor 3 Diet adherence**	**Factor 4 Healthy lifestyle adherence**
C5	.813	.056	.136	.226
C3	.783	.139	.086	.337
C2	.773	.127	.143	.224
C4	.758	.046	.223	.172
C1	.745	.114	.159	.271
C6	.744	.064	.154	.204
C7	.695	.088	.116	.039
B2	.111	.939	.121	.113
B3	.124	.919	.137	.159
B4	.118	.884	.100	.191
B1	.088	.864	.148	.147
B5	.078	.672	.169	.258
A6	.116	.120	.872	.174
A5	.123	.167	.852	.161
A4	.118	.161	.843	.135
A3	.196	.071	.741	.089
A7	.281	.134	.691	-.034
D8	.195	.027	.017	.664
D2	-.047	.233	.099	.651
D11	.190	.221	.076	.650
D9	.224	.182	.073	.642
D7	.364	.036	.037	.612
D6	.228	.092	.194	.562
D1	.200	.135	.102	.516
**Variance explained by each factor(%)**	19.187	16.785	14.774	13.597
**Eigenvalues of each factor**	4.605	4.028	3.546	3.263

Extraction method: Principal component analysis Rotation method: Varimax with Kaiser normalization

### CFA of the 24-item HEAS-SP

**Fig. 1:**
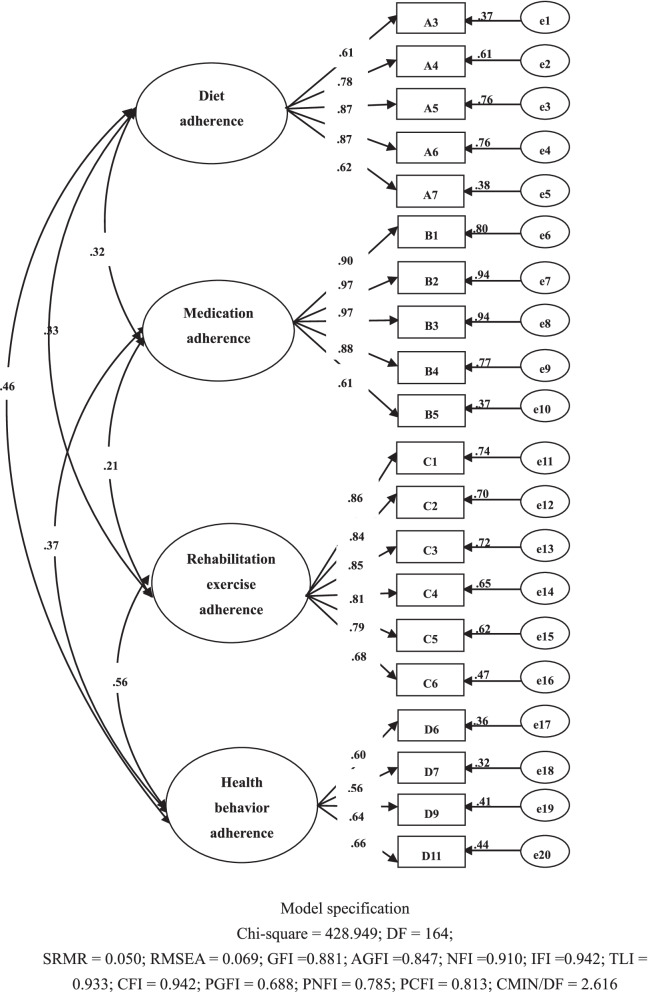
Model structure and standardized factor loadings of the four-factor model of the 20-item HEAS-SP (*N* = 331)

The other half of the dataset, containing data from the group of 331 participants, was used to run CFA to further confirm the factor structure of the 24-item HEAS-SP. After the first round of CFA, four items were deleted due to standardized factor loadings of less than 0.55: items D1, D2, D8, and C7.The final CFA with the 20-item HEAS-SP confirmed the four-factor structure obtained via EFA. All of the model fit indexes met the criteria for a good model (See model specification in Fig. 1). In addition, all of the standardized factor loadings of items were** ≥ **0.55, the variance explained by each item ranged from 32 to 94%, and the correlations among the four domains ranged from 0.21 to 0.56 (Fig. 1).

### Reliability of the 20-item HEAS-SP

The Cronbach’s α of the 20-item HEAS-SP was 0.90, and the Cronbach’s α values for the four domains of diet adherence, medication adherence, rehabilitation exercise adherence and healthy lifestyle adherence were 0.87, 0.90, 0.92, and 0.71, respectively. The corrected item-total correlations were 0.41–0.64, and the item-subscale correlations were 0.46–0.92 (Table 4). The above three types of internal reliability coefficients all met the criteria that were set, indicating good reliability of the 20-item HEAS-SP. Furthermore, the Pearson correlation coefficient of the overall HEAS-SP scale was 0.92, and the coefficient for the four domains of diet adherence, medication adherence, rehabilitation exercise adherence and healthy lifestyle adherence were0.96, 0.84, 0.86, and 0.82, respectively. All the coefficient exceeded the criteria of 0.70, indicating acceptable test–retest reliability of the HEAS-SP.

**Table 4:** Internal reliability of the 20-item HEAS-SP (*N* = 331)

Items	Corrected item-total correlation	Item-subscale Correlation	Cronbach’s α	M ± SD	Range
C1	0.621	0.792	0.916	18.89 ± 6.21	6–30
C2	0.592	0.770			
C3	0.638	0.801			
C4	0.559	0.780			
C5	0.575	0.774			
C6	0.565	0.665			
B1	0.504	0.824	0.901	23.34 ± 5.79	5–25
B2	0.550	0.907			
B3	0.570	0.912			
B4	0.550	0.873			
B5	0.466	0.611			
A3	0.405	0.604	0.867	17.01 ± 3.94	5–25
A4	0.496	0.747			
A5	0.529	0.756			
A6	0.524	0.768			
A7	0.489	0.584			
D6	0.429	0.485	0.709	12.45 ± 3.47	4–20
D7	0.408	0.462			
D9	0.455	0.506			
D11	0.475	0.538			
Total scale			0.896	69.45 ± 13.21	20–100

### Validity of the 20-item HEAS-SP

The CR of the total HEAS-SP is 0.97 and the CR is from 0.71 to 0.94 for the four domains (Table 5), and the AVEs of each domain of the HEAS-SP is from 0.388 to 0.58. The above results indicated acceptable convergent validity of the HEAS-SP. Besides, all the square root of the AVEs of each domain are greater than the correlation coefficient of each counterpart domain and other domains (Table 5), which is evidence of acceptable discriminant validity of the HEAS-SP.

**Table 5:** Composite reliability (CR), Estimated correlations between domains and average variance extracted (AVE) of each domain (*N* = 331)

Domains	CR	AVE	Diet adherence	Medication Adherence	Rehabilitation Exercise Adherence	Healthy Lifestyle Adherence
Diet adherence	0.869	0.576	**0.759**			
Medication Adherence	0.942	0.767	0.304^**^	**0.876**		
Rehabilitation Exercise Adherence	0.918	0.652	0.280^**^	0.184^**^	**0.807**	
Healthy Lifestyle Adherence	0.709	0.378	0.332^**^	0.250^**^	0.445^**^	**0.615**

^**^ *p* ≤ 0.01, Square root of AVE in bold diagonals

## Discussion

### The item reduction procedure

The item analysis, EFA and CFA employed for item reduction yielded a 20-item HEAS-SP. Specifically, the item analysis, the EFA and the CFA deleted 2, 8 and 4 items, respectively. The final CFA yielded a four-factor structure for the 20-item HEAS-SP with good model fit indexes. The three item reduction process has been adopted by other researchers and was proven to be a rigorous process for item selection [23, 32, 33].

In the item analysis, items A1 and A2 in the diet adherence domain were removed for not qualifying on 3 indicators, which means that the two items may not be applicable for measuring stroke patients’ adherence in diet. Specifically, item A1 emphasizes the patients’ adherence to the type of diet according to their swallowing condition, however, the stroke patients may feel difficult to evaluate their swallowing condition by themselves and thus this item perform not well on the item analysis. In addition, item A2 measures the patients’ adherence to simple swallowing training method, it is actually not directly related to the diet adherence, thus it performed poor on item analysis.

In the 4 rounds EFA, eight items were removed in sequence: B8, D3, D4, A8, D10, B6, B7, and D5. Specifically, item B8 in the medication adherence domain was removed in the first round of EFA because it did not significantly load on any domain. Item B8 focuses on the patients’ adherence to paying attention to their blood pressure when taking medication. This item is not directly related to medication adherence, and thus makes it perform poorly on the EFA. Items D3 and D4 in the healthy lifestyle adherence domain, and item A8 in the diet adherence domain were removed in the second round of EFA because the three items loaded on one separate domain, which was not easy to explain from the clinical perspective. Moreover, some other reasons may cause the abnormal performance on EFA for the three items. Item D3 assesses the patients’ adherence to doing rehabilitation exercises on time, but this item seems to be too abstract for stroke patients in that it didn’t indicate specific types of rehabilitation exercises, which made it not well understood by the patients and thus performed poorly in the EFA. Item D4 evaluates the patients’ adherence to mastering stroke-related factors. There are many risk factors related to stroke and each participant’s situation is different. Multiple risk factors in the item cause the patient to be confused, so they cannot make the right or appropriate choice on this item. Item A8 measures the patients’ adherence to controlling the amount of food they eat. Though it is closely related to diet adherence, it is not significantly loaded on the diet adherence domain and thus was deleted according to the statistical rules for EFA. Item D10 in the healthy lifestyle adherence domain was removed in the third round of EFA. Item D10 was designed to evaluate the patients’ adherence to regularly monitoring their glycated hemoglobin, which was attributed to the healthy lifestyle adherence domain by the researchers according to its clinical significance. However, it loaded on the medication adherence domain. The reason may be that the medical term “glycated hemoglobin” may be misunderstood by the stroke patients as the name of one medication, which makes this item wrongly loaded on the medication adherence domain. Items B6 and B7 in the medication adherence domain, and item D5 in the healthy lifestyle adherence domain were removed in the fourth round of EFA. Items B6 and B7 loaded as a separate domain since they were not directly related to the medication taking. Specifically, items B6 and B7 seek to assess the patients’ adherence to monitoring the adverse reactions of the medicine and following the ASA (hypertensive drug—lipid-lowering drug—anticoagulant drug) drug treatment strategy. However, this two health problems may be ignored by the stroke patients for that the two items were too professional-oriented to be followed. Therefore, items B6 and B7 were difficult for the patients to comply with and were deleted. Items D5 loaded on the rehabilitation exercise domain in the fourth round EFA, which was reasonable and easy to understand as it aimed to assess the patients’ adherence to physical activity. However, items D5 was similar to the item “I can adhere to the nurses’ health education to exercise on time every day” in the rehabilitation exercise domain. Thus, we deleted item D5 following the minimalistic principles of measurement development.

Four items were removed in the CFA, which were item C7 in the rehabilitation exercise adherence domain and items D1, D2, and D8 in the healthy lifestyle adherence domain. These items were removed because their standardized factor loadings were lower than the criteria of 0.55, which explained less than 30.25% (0.55^2^) of the total adherence variance. The lower variance explained by the above items indicated that these items were not well reflective of the health education adherence, and the deletion of the four items makes the final 20-item model perfect. Thus, in order to choose the items that best reflect the health education adherence of stroke patients, items C7, D1, D2, and D8 were deleted to gain the most simplified and best model.

### Factor structure, reliability and validity of the 20-item HEAS-SP

The item reduction process yielded a 20-item HEAS-SP with four domains: diet adherence (5 items), medication adherence (5 items), rehabilitation exercise adherence (6 items) and healthy lifestyle adherence (4 items).All the items retained in the final scale explained 69.1% of the total health education adherence variance, indicating that the 20-item model is highly representative of the health education adherence of stroke patients [28]. Diet adherence refers to stroke patients’ adherence with respect to eating methods, diet type and eating behaviors, etc. Medication adherence refers to stroke patients’ adherence with respect to medication methods, number of medications, and medication types, etc. Rehabilitation exercise adherence refers to stroke patients’ adherence with respect to rehabilitation exercise methods and rehabilitation start time and duration, etc. Healthy lifestyle adherence refers to stroke patients’ adherence with respect to lifestyle, risk factor monitoring, living conditions, emotional control and follow-up time, etc. The four domains of the 20-item HEAS-SP comprehensively cover the content of health education for stroke patients, and provide an effective tool for monitoring the adherence of health education for stroke patients.

The three internal reliability indicators of Cronbach’s α, corrected item-total correlation and item-subscale correlation met the standard criteria for acceptable reliability, which indicated that the 20-item HEAS-SP is a reliable scale for assessing health education adherence in stroke patients. And the Pearson correlation coefficient met the standard for acceptable test–retest reliability indicating that the result of HEAS-SP was stable over time. In addition, all the domain AVEs met the standards for acceptable convergent validity except for the healthy lifestyle adherence domain (AVE = 0.378); and all the CR were ≥ 0.70, which is also evidence of acceptable convergent validity. Notably, although the AVE of the healthy lifestyle adherence domain is less than 0.50, according to some researcher that if AVE is less than 0.50 but composite reliability is higher than 0.60, the convergent validity of the scale is still adequate [34]. Furthermore, the square root of AVEs of each domain is more than the correlation coefficient of each counterpart domain and other domains, which indicated acceptable discriminant validity of the HEAS-SP.

### Clinical interpretability of the HEAS-SP

The 20-item HEAS-SP is a reliable and valid assessment tool for clinical health care workers, and could facilitate the assessment of the health education adherence of stroke patients and the formulation of individualized health education plans for stroke patients. Compared with the current health education adherence scales, most of which assess only one aspect of health education adherence, the 20-item HEAS-SP is more comprehensive because it includes four aspects of the health education adherence of stroke patients, namely, diet, medication, rehabilitation exercise, and healthy lifestyle. Currently, there is no scale for assessing health education adherence with respect to diet. This study developed specific items in diet for stroke patients that accurately reflect their adherence to diet health education. Compared with the most commonly used scale to assess medication adherence, the MMAS-8 [16], the medication adherence domain in our scale was more targeted via the two items on adhering to medication methods and medication type specific for stroke patients. The primary scale currently used to assess health education adherence with respect to rehabilitation exercise is the EAQ [19], but it may not be suitable for evaluating health education adherence with respect to rehabilitation exercise for hospitalized stroke patients. The rehabilitation exercise adherence domain in the 20-item HEAS-SP is more widely applicable for both hospitalized and community stroke patients. Currently, there is no scale to assess health education adherence with respect to healthy lifestyle adherence. We included a healthy lifestyle adherence domain in the 20-item HEAS-SP, which can be used to effectively assess healthy lifestyle adherence in stroke patients. The four domains of the HEAS-SP—diet, medication, rehabilitation exercise and healthy lifestyle adherence—can comprehensively assess the health education adherence of stroke patients and thus provide a basis for the development of targeted health education programs.

### Limitations of this study

This study has several limitations. First, a statistical driven approach was used for item selection, we may delete items that are representative for health education adherence. Future research is planned to further explore the multi-faceted construct of the 20-item HEAS-SP. Second, although the 20-item HEAS-SP was shown to be a reliable and valid measurement according to the excellent numerical ranges on the statistical methods, future empirical research are needed to further confirm its usefulness in various clinical settings. Third, the number of hospitalizations of the participants was not included in the demographic questionnaire. As the number of hospitalizations may affect the compliance of the patients with health education, we will take this variable into account in our future health education research. Fourth, we have not modified the HEAS-SP at the national, regional and community level, future research is on the way to modify the scale at different levels.

## Conclusions

The 20-item HEAS-SP was shown to have acceptable reliability and validity for assessing health education adherence with respect to diet, medication, rehabilitation exercise and healthy lifestyle in stroke patients, making it a potential basis for developing targeted interventions for stroke patients.

## Data Availability

The datasets used and/or analyzed during the current study are available from the corresponding author on reasonable request.

## Abbreviations

HEAS-SP: Health Education Adherence Scale for Stroke Patients
MMAS-8: Morisky Medication Adherence Scale
EAQ: Questionnaire of Exercise Adherence
MMSE: Mini-Mental State Examination score
EFA: Exploratory factor analysis
CFA: Confirmatory factor analysis
I-CVI: Item-level content validity index
S-CVI/Ave: Average scale-level content validity index
PCA: Principal component analysis
KMO: Bartlett’s test of sphericity and the Kaiser–Meyer–Olkin
SRMR: Root mean square residual
RMSEA: Root-mean-square error of approximation
GFI: Goodness of fit index
AGFI: Adjusted GFI
NFI: Normed fit index
IFI: Incremental fit index
TLI: Tucker-Lewis index
CFI: Comparative fit index
PGFI: Parsimony goodness-of-fit index
PNFI: Parsimony adjusted NFI
PCFI: Parsimony comparative fit index
CMIN/DF: Chi-square value/degrees of freedom
CR: Composite reliability
AVE: Average variance extracted
